# Multimode OAM beam generation through 1-bit programmable metasurface antenna

**DOI:** 10.1038/s41598-023-42691-0

**Published:** 2023-09-20

**Authors:** Morteza Nadi, Seyed Hassan Sedighy, Ahmad Cheldavi

**Affiliations:** 1 School of Electrical Engineering, Iran University of Science and Technology, Tehran, Iran; 2 School of Advanced Technologies, Iran University of Science and Technology, Tehran, Iran

**Keywords:** Metamaterials, Electrical and electronic engineering

## Abstract

modern wireless communication, the orbital angular momentum (OAM) beam is considered as an important technology. Some considerable efforts have been devoted to using this technology for channel capacity enhancement as much as possible. Nowadays, programmable metasurfaces provide an innovational scenario for generating multi-mode OAM beams due to their ability for digital electromagnetic waves modulation. However, the current programmable metasurfaces for generating OAM beams are typically based on reflective and transmissive modes, which have low aperture efficiency due to spillover and illumination effects. In this paper, a 1-bit programmable metasurface antenna is proposed with capability of producing highly efficient dynamic multi-mode OAM beams. The proposed structure is consisted of electronically reconfigurable meta-radiating elements loaded by PIN diodes to generate two-phase states of electric field. The designed Field Programmable Gate Array (FPGA) can assign a code sequence of 0 or 1 to the metasurface antenna in real-time to generate multi-mode OAM beams. Hence, a dynamical surface is obtained by switching PIN diodes to change the phase distribution on the surface. To verify the concept, the metasurface antenna is fabricated and measured with different OAM beam states, which are in agreement with the full-wave simulations, properly. The designed structure introduces a capable multi-mode OAM alternative for high throughput mm-wave communications.

## Introduction

The infrastructure development to accommodate data traffic demands are resulted in wireless technologies migration from fourth-generation (4G) to fifth-generation (5G). As compared to 4G, 5G is expected to increase aggregate data rates by 1000 times^1^. The 5G and 5G-beyond are expected to improve spectrum efficiency through its feature, which include massive multiple input multiple output (MIMO), co-frequency and co-time full-duplex, as well as using millimeter wave (mm-Wave). For this purpose, a variety of orthogonal resources have been extensively explored in the recent decades, including frequency, time, and space. In today's world, it is becoming increasingly hard to support more users or increase capacity using traditional access techniques such as time-division-multiple-access (TDMA), and frequency-division-multiple-access (FDMA)^2^. Therefore, introducing alternative methods are more prevalent than before. With the introduction of angular momentum beams such as spin angular momentum (SAM) and orbital angular momentum (OAM), it is possible to attain enormous gains in spectrum efficiency and channel capacity^3^. There are a great number of topological charges in OAM, referring to OAM-modes, which have attracted a lot of research attentions. Due to the orthogonal nature of OAM modes, beams can be multiplexed and demultiplexed, and capacity is maximized consequently, without relying on traditional resources like time and frequency. In fact, it would be possible to significantly improve spectrum efficiency in future networks including 5G and beyond by using multiple orthogonal topological charges in OAM^4^.

Many elegant design approaches have been proposed for the generation of OAM carrying beams with helical transverse phase structure of $exp\left(il\varphi \right)$ (in which $l$ is the OAM topological charge, and $\varphi$ is the azimuth angle^5–20^) including reconfigurable array antennas^6^, circular phased arrays^7,8^, and metasurfaces^9–14^. Specifically, the programmable metasurfaces are reconfigurable planar structures which can manipulate electromagnetic waves in an unprecedented way^15–20^.

The metasurfaces are two-dimensional equivalence of a metamaterials which attracting more and more attention within both science and engineering communities, due to its effective and intriguing applications such as scattering reduction^21–23^, reflectarray and transmitarray antenna^24–26^, Intelligent surface^27,28^, invisibility clocking^29,30^, and polarization conversion^31^. Also, the metasurfaces ability to manipulate electromagnetic waves can be used to produce vortex beams in both radio and optics frequency. Many studies have explored the development of broadband vortex beams, multiple OAM modes, and reconfigurable OAM modes using active components on transmissive or reflective metasurfaces. A programmable 1-bit reflective metasurface was introduced to stimulate multi-mode mm-Wave vortex beams, dynamically by integrating PIN diodes^32^. The multifunctional X-band vortex beams have been generated by manipulating the vortex phase fronts in both space and time using a programmable 3-bit reflective metasurface in^33^. Also, the transmission of dynamical multi-mode vortex beams was introduced to avoid feed blocking in reflective mode^34,35^.

It is worth noting that the majority of structures that generate vortex beams are used to control the incident wave, while they have only a limited number of practical applications. As a solution to overcome the limitation of this technology in cellular communications, here we propose a planar programmable metasurface antenna for generating dynamically OAM-beams at microwave frequencies.

Metasurface antennas are currently being developed as an effective way to manipulate electromagnetic waves by enabling meta-radiates for feeding themselves without needing of external illumination such as horn antennas^36,37^. Unlike conventional array antennas with half wavelength spacing between elements, the radiating elements of these structures are placed in less space. As a result, more radiating elements can be accommodated in the same footprint resulting in a more efficient aperture and higher gain, consequently.

**Figure 1:**
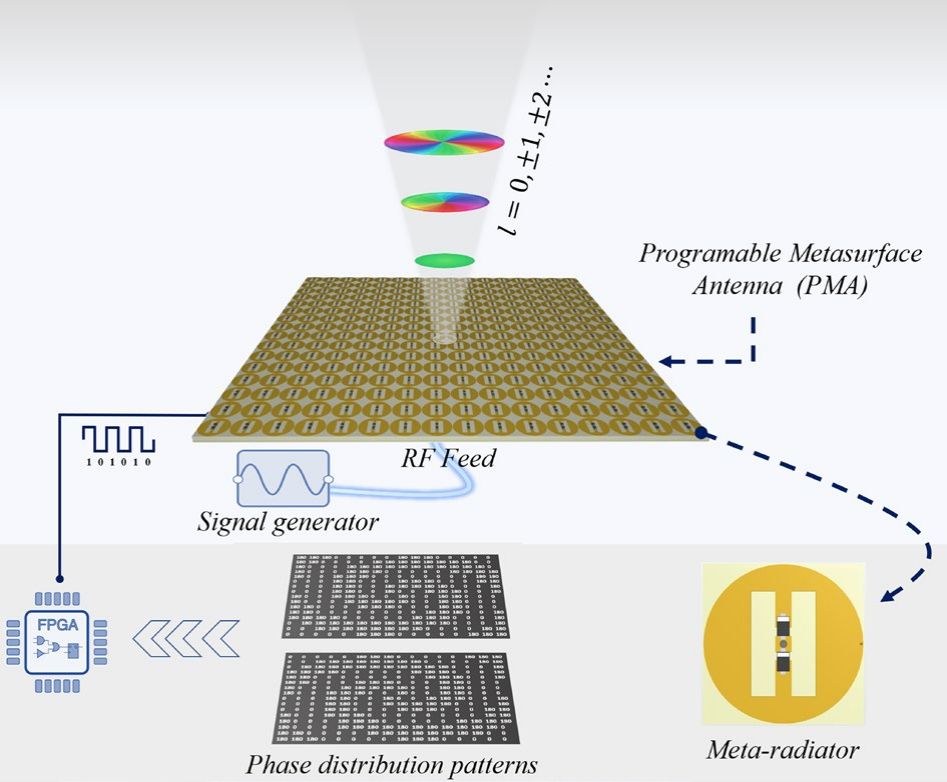
Topology of the programmable mm-Wave PMA for dynamic OAM beam generation.

In this paper, a programmable metasurface antenna (PMA) is introduced for stimulating dynamic multi-mode mm-Wave vortex beams. For this propose, a geometrically-fixed array antenna is proposed which is composed of meta-radiating elements loaded by PIN diodes. The overall schematic function of the proposed PMA is presented in Fig. 1. The creation of dynamic multimode mm-Wave vortex beams can be achieved by modulating the real-time coding distribution of a metasurface using a programmable bias circuit. The bias circuit changes the phase state of each radiator by applying the correct voltage, where one of the diodes is always on. Each radiator element is excited by a pin connected to a simple corporate feed network with phase states (0/ π) through switching PIN diodes. Additionally, as a proof of concept, a dynamic programmable metasurface antenna operating at 6 GHz with 16 × 16 units is designed and fabricated which can generate three-mode adjustable vortex beams, including OAM modes $l=0$, $l=\pm 1$ and $l=\pm 2$. Both simulations and experiments confirm the generation of these three OAM beams, which is proof of our design's effectiveness. The OAM metasurface antenna with multi-modes is suitable for future mm-Wave and multi-mode OAM hybrid communications.

## Theoretical formulation

This section presents the theoretical formulation of a PMA performance in generating multi-mode OAM beam by applying vortex beam and array antenna theory. In this analysis, we consider a metasurface periodic array antenna in the transverse x–y plane consisting of subwavelength meta-radiators programming by control board, under the excitation of microwave signal at the operating frequency, ${f}_{0}$. The far field function radiated by the metasurface antenna is expressed as

1 $$F=\sum_{m=1}^{M}\sum_{n=1}^{N}{I}_{mn}{e}^{jk{\overrightarrow{r}}_{mn}.{\widehat{a}}_{r}}$$

where ${\overrightarrow{r}}_{mn}$ is a position vector to the (*m, n*)th element, ${\widehat{a}}_{r}$ is a unit vector pointing in the direction of interest, and ${I}_{mn}={A}_{mn}{e}^{j{\varphi }_{mn}}$. In order to stimulate converged OAM beams at the PMA, each meta-radiator requires theoretical phase $\varphi$ as follows

2 $${\varphi \left(m,n\right)}_{OAM}=l\times arctan\left(\frac{y}{x}\right)+P.P$$

where $l$ is the OAM mode, $x$ and $y$ are the position of meta-radiator in the Cartesian coordinate, and P.P is progressive phase shift, which is ($P.P=\frac{2\pi }{\lambda }\sqrt{{x}^{2}+{y}^{2}}$), $\lambda$ is the wavelength at operating frequency. Moreover, since the reconfigurable meta-radiator can only display binary phase states, the phases should be quantized into two coding states as follows

3 $${\varphi \left(m.n\right)}_{QOAM}=\left\{\begin{array}{c}0, {\varphi }_{OAM}\in \left[\left.0+2n\pi , \pi +2n\pi \right) \right.\\ \pi , {\varphi }_{OAM}\in \left[\left.\pi +2n\pi , 2\pi +2n\pi \right) \right.\end{array}\right.\mathrm{ n}\in \mathrm{Z}.$$

The progressive signal phase is compensated by means of a planar transmission line network. As a result of this method, spillover and illumination losses are no longer, also. It is worth noting that the progressive phase is identical for all OAM modes, resulting in one identical feed network.

This theory illustrates the capability of PMA in increasing capacity and improving spectrum efficiency by generating multi-mode OAM beams. It should be mentioned that while the binary phase distribution on the surface antenna is changed by control board, the OAM-beams can be radiated which are orthogonal with each other.

## Meta-radiator design

**Figure 2:**
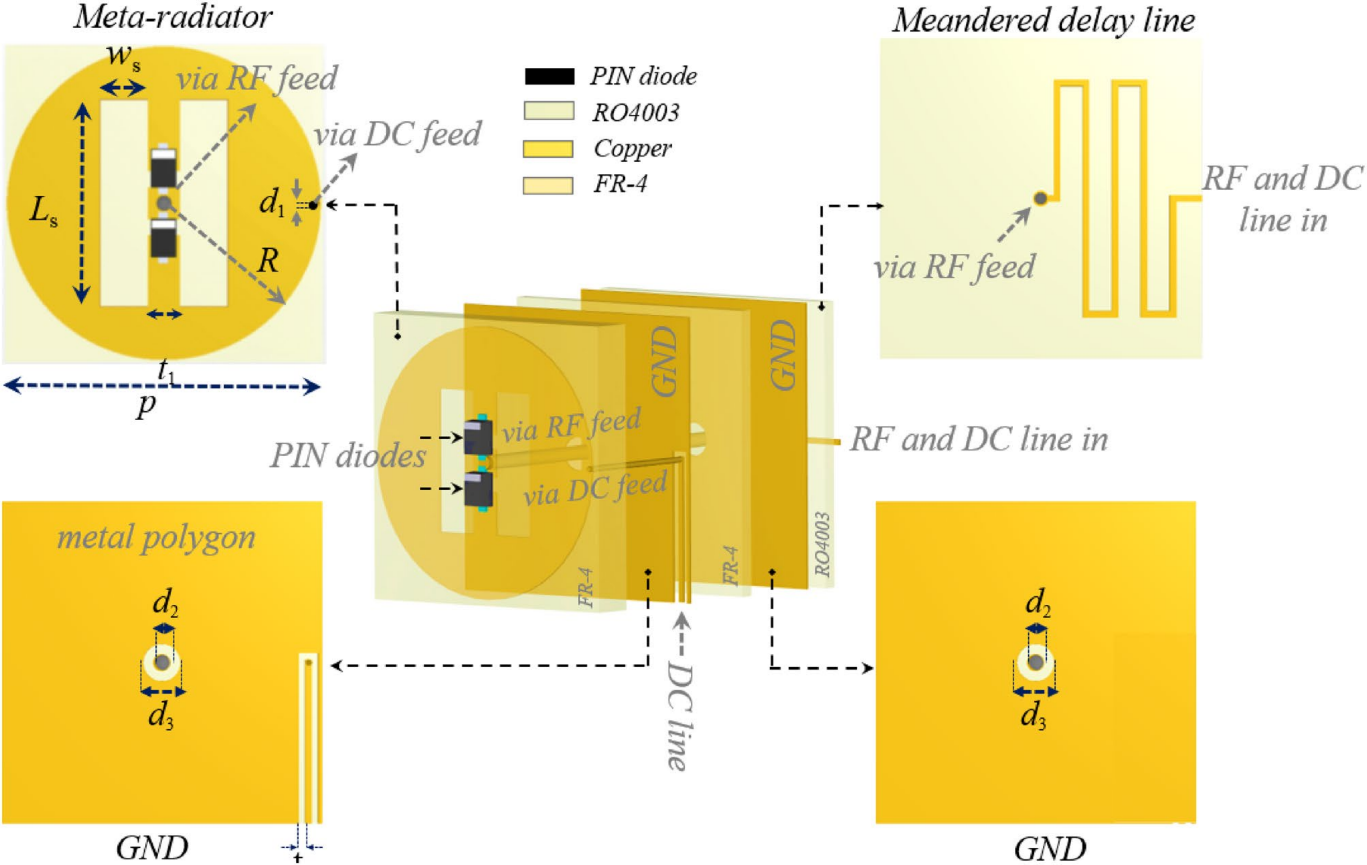
Design details and parameters of the binary meta-radiator for 1-bit PMA.

In order to demonstrate the applicability of the multi-mode OAM-beam metasurface antenna concept for increasing capacity and improving spectrum efficiency, as well as numerically verification of the design concept formulated in the previous section, we design and model a programmable metasurface antenna by utilizing PIN diodes as its constituent binary meta-radiator. For this purpose, the meta-radiator is designed with four metal layers separated by three substrates, as shown in Fig. 2. The radiator layers are arranged from top to bottom as follows: (1) a metallic radiator with an electrically small periodicity of p = 10 mm is ingeniously printed on the top side of a commercially available FR-4 substrate $\left({\varepsilon }_{r}=4.3, tan\delta =0.025\right)$ with h = 1.6 mm thickness $\left(0.03{\lambda }_{0}\right)$, (2) the biasing line are printed on FR-4 layer also, with h = 0.5mm thickness while being fed by distinct digital DC voltages; low and high, (3) the copper ground plane with a conductivity of σ = 5.7 × 107 S/m printed on Rogers 4003C $\left({\varepsilon }_{r}=3.55, tan\delta =0.0027\right)$ with h = 0.5mm thickness. The last metal layer is a corporate microstrip-based feeding network underneath the ground plane. In this layer, a meandered line produces the progressive phase necessary for OAM beam radiation. This phase depends on where the meta-radiators are located.

It is important to mention that the feed network is placed below the ground plane to prevent negative effects of radiation performance and feeding efficiency. The energy is transferred from feed network to meta-radiators via metallized via holes located at the center of meta-radiator. There are three voltage levels used to control the radiator, named low, mid, and high voltage. Among these three voltage levels, the middle voltage level is injected into the RF line by a bias tee circuit which is served as the reference voltage. Through bias lines, two other voltage levels (low and high) are applied to the meta-radiator by switching between them with the control board. For minimizing the impact of the bias line on radiation performance, a metal polygon is used to cover the bias layer. The meta-radiator is connected to the bias layer at the point where the current is almost zero, which occurs on the right or left side of the radiator. The bias lines have a very narrow linewidth (t_2_ = 0.2 mm) and are placed very close to the metal polygon. Additionally, they are lined up symmetrically beneath the radiating layer to connect with the FPGA from the antenna side.

**Figure 3:**
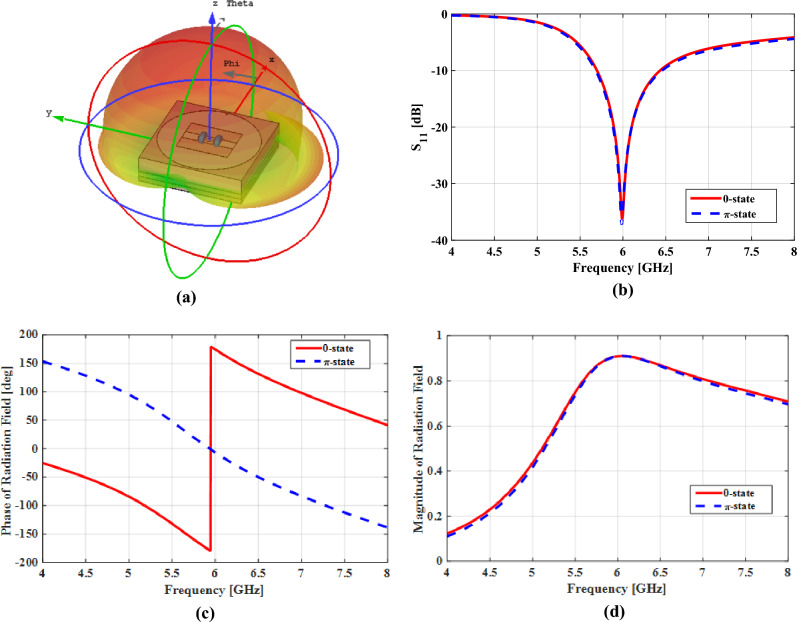
(**a**) The element pattern function and (**b**) return loss of the designed meta-radiator. (**c**) The phase and (**d**) amplitude of the 0 and π sate electric fields radiated by the designed binary meta-radiator.

The binary meta-radiator in Fig. 2 is composed of two embedded PIN diodes that connect the feeding pin to the radiated patch, and works in both "ON" and "OFF" modes in which surface current can only pass through one of the diodes and radiate, consequently. As the current passing through the diodes is in opposite directions, there is a 180-degree phase difference in the electric field radiated by the meta-radiator, also. It should be noted that since the radiators are designed by using periodic boundary conditions, their dimensions are the same as the radiator periodicities, which are smaller than an operative wavelength of approximately $0.2{\lambda }_{0}\left(f=6 \mathrm{GHz}\right)$. In other words, the coupling effect between the adjacent elements is characterized by periodic boundary conditions. Therefore, the footprints of the metasurface antenna are smaller than those of conventional arrays made up of sparse antenna elements. In the study at the microscopic level, each of the binary meta-radiators is excited from its termination by a lumped port with an internal impedance equal to the impedance matching value that is calculated from simulation (100 Ω). The geometrical parameters are W_s_ = 1.5 mm, L_s_ = 6.5 mm, R = 4.9 mm, and t_1_ = 1 mm. The numerical simulation is performed with CST Microwave Studio, with periodic boundary conditions along x and y directions, where Floquet ports is assigned in z direction. A series circuit is used to model pin diodes in the simulated model, by resistance ${R}_{on}=0.85\Omega$ and inductance ${L}_{on}=0.45 \mathrm{nH}$ in turned on mode, and resistance ${R}_{off}=5 \mathrm{M\Omega }$, capacitance ${C}_{off}=0.21 \mathrm{pF}$ and inductance ${L}_{off}=0.45 \mathrm{nH}$ in turned off mode. According to Fig. 3a, the employed meta-radiator produces a normal and symmetric radiation pattern along the broadside direction at *f* = 6 GHz. Also, the phase and amplitude spectra of radiation electric fields in various states of PIN diodes are depicted in Fig. 3c, d, respectively. As it can be seen, a constant 180 phase difference with an amplitude greater than 0.9 is successfully obtained, at the operating frequency. Notice that the less than unity amplitude of the designed binary meta-radiators is resulted from the diode ohmic loss. As shown in Fig. 3b, the return loss curve of the binary meta-radiators is below 10 dB (|S11|< −10 dB) at operating frequency for different states of PIN diodes.

## OAM metasurface antenna design

**Figure 4:**
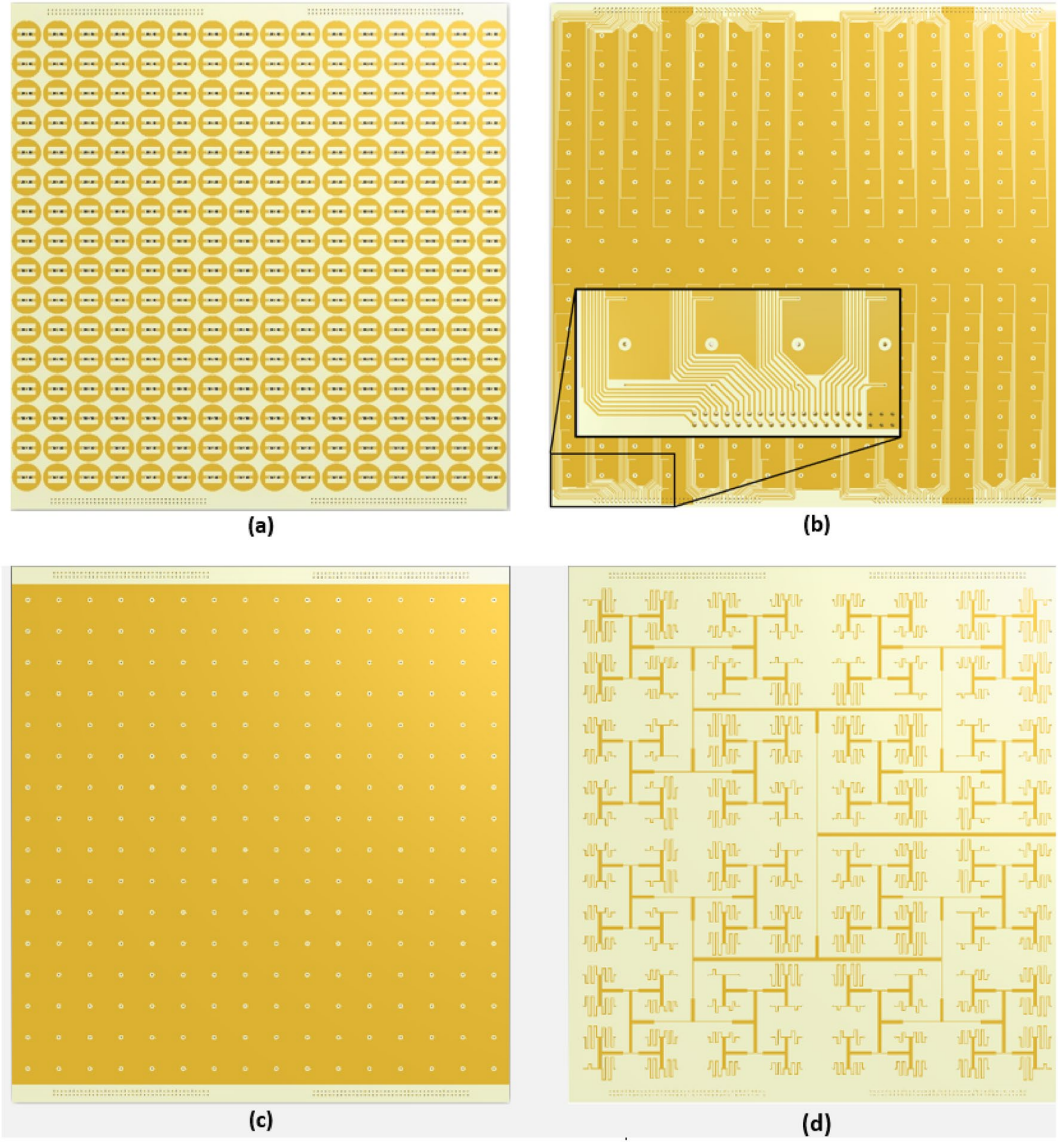
Overall structure of the proposed PMA with 16 × 16 binary meta-radiators. (**a**) Top layer with the binary meta-radiators. (**b**) Bias layer with the bias network layout. (**c**) Ground plane. (**d**) Microstrip feed network layer.

In order to corroborate the ability of PMAs to control the spectral diversity of OAM beams, we develop a metasurface antenna based on a binary meta-radiator discussed in the previous section. For this purpose, the PMA is designed which is composed of 16 × 16 meta-radiators with the effective dimension of 3.2 λ0 × 3.2λ0 at the central frequency, 6 GHz. Figure 4 shows the overall configuration of the proposed PMA, in which meta-radiators along with 512 PIN diodes are utilized. The bias layer network is composed of four symmetrical parts to avoid interference between them as shown in Fig. 4b. The bias and RF feed network are isolated by the ground plane, as shown in Fig. 4c, also. According to Fig. 4d, a corporate microstrip feeding network is used to stimulate the top side meta-radiators. It should be noted that that the progressive phase obtained from Eq. (2) can be simply implemented by adding meandered transmission lines.

**Figure 5:**
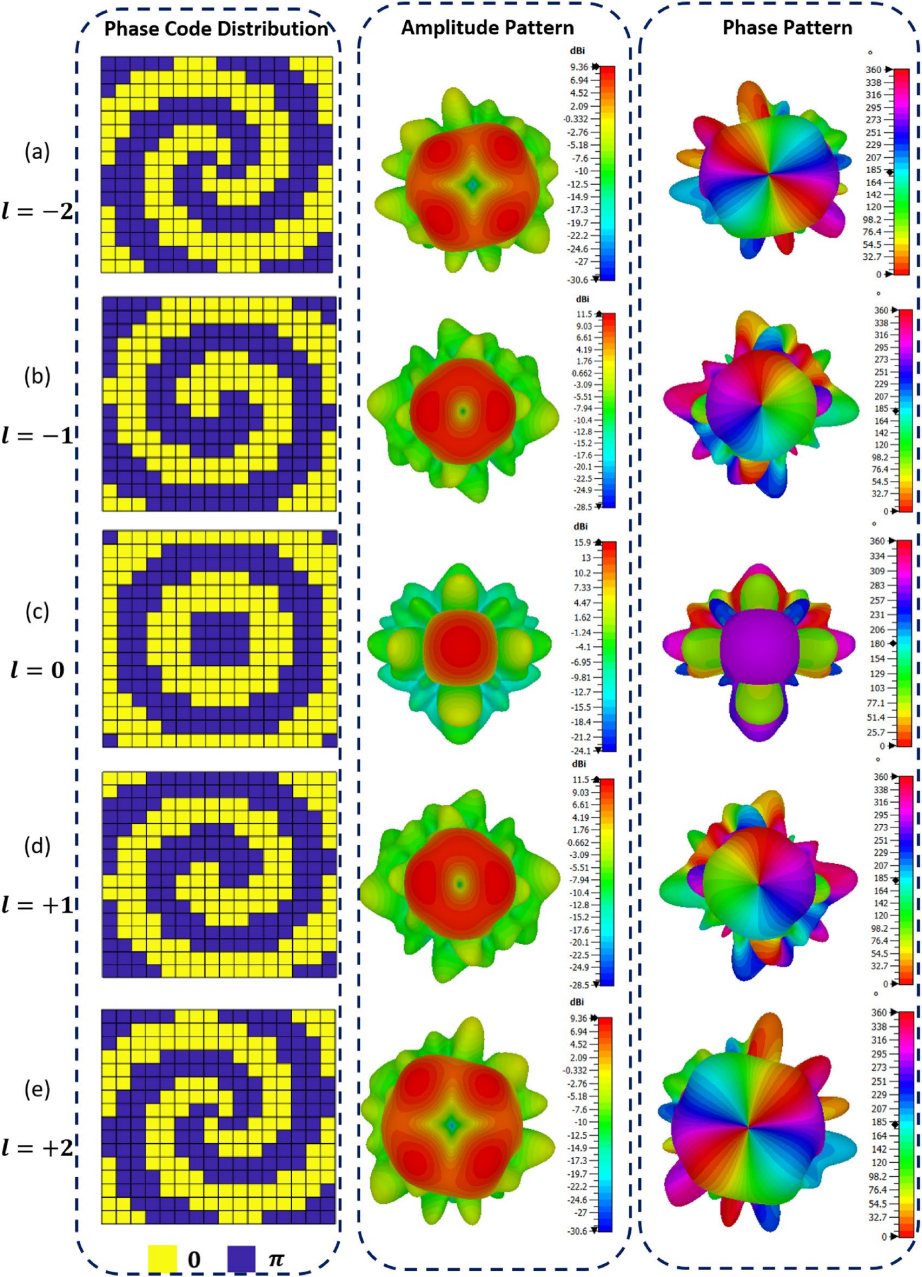
Distributions of codes and patterns of simulated radiation for various modes of OAM: (**a**) OAM mode $l=-2$, (**b**) $l=-1$, (**c**) $l=0$, (**d**) $l=+1$ and (**e**) $l=+2$.

On the basis of the PMA, Initial numerical simulations are performed by using four generations of convergent vortex beams, including OAM modes $l=0$, $l=\pm 1$, $l=\pm 2$, as shown in Fig. 5. According to Eq. (2), the required quantized code distribution for the first three modes is shown in the first column of Fig. 5, also. It is clearly evident that all phase distributions except the first mode (i = 0) have vortex configurations. On the second column of Fig. 5, the simulated amplitude patterns of far-field radiation for the three OAM modes are presented. High intensity conical patterns clearly show up for modes other than zero, while a high gain directional pencil beam is clearly visible for zero mode $\left(l=0\right)$. Based on the third column of Fig. 5, it is evident that the simulated far-field radiation phase patterns are characterized by vortex-shaped phase fronts and on-axis phase singularities for OAM modes except zero mode. It is also possible to obtain the radiation characteristics of the two negative-mode OAM beams, whose phase profiles are in direct opposition to positive-mode OAM beams according to the conventional mirror image principle.

Moreover, the vortex waves with their helical phase structure are capable of transporting OAM. The phase singularity causes the electromagnetic wavefront to twist during propagation, resulting a dark spot in the propagation direction. Moreover, each vortex has a number and sign of OAM-modes which indicate the number of wave torsions within one wavelength and the chirality of the wave, respectively. When the number of twists increases, the wave is rotating faster around the axis. OAM waves with different modes, independent of frequency, time, and polarization, can add a new dimension to data transmission due to the inherent orthogonality between OAM modes, thereby dramatically improving spectrum efficiency. This method with these unique properties makes it a good candidate to be used in massive multiple input multiple output (MIMO) technology that is going to be one of the key technologies for 5G or beyond 5G networks.

## Experimental verifications

**Figure 6:**
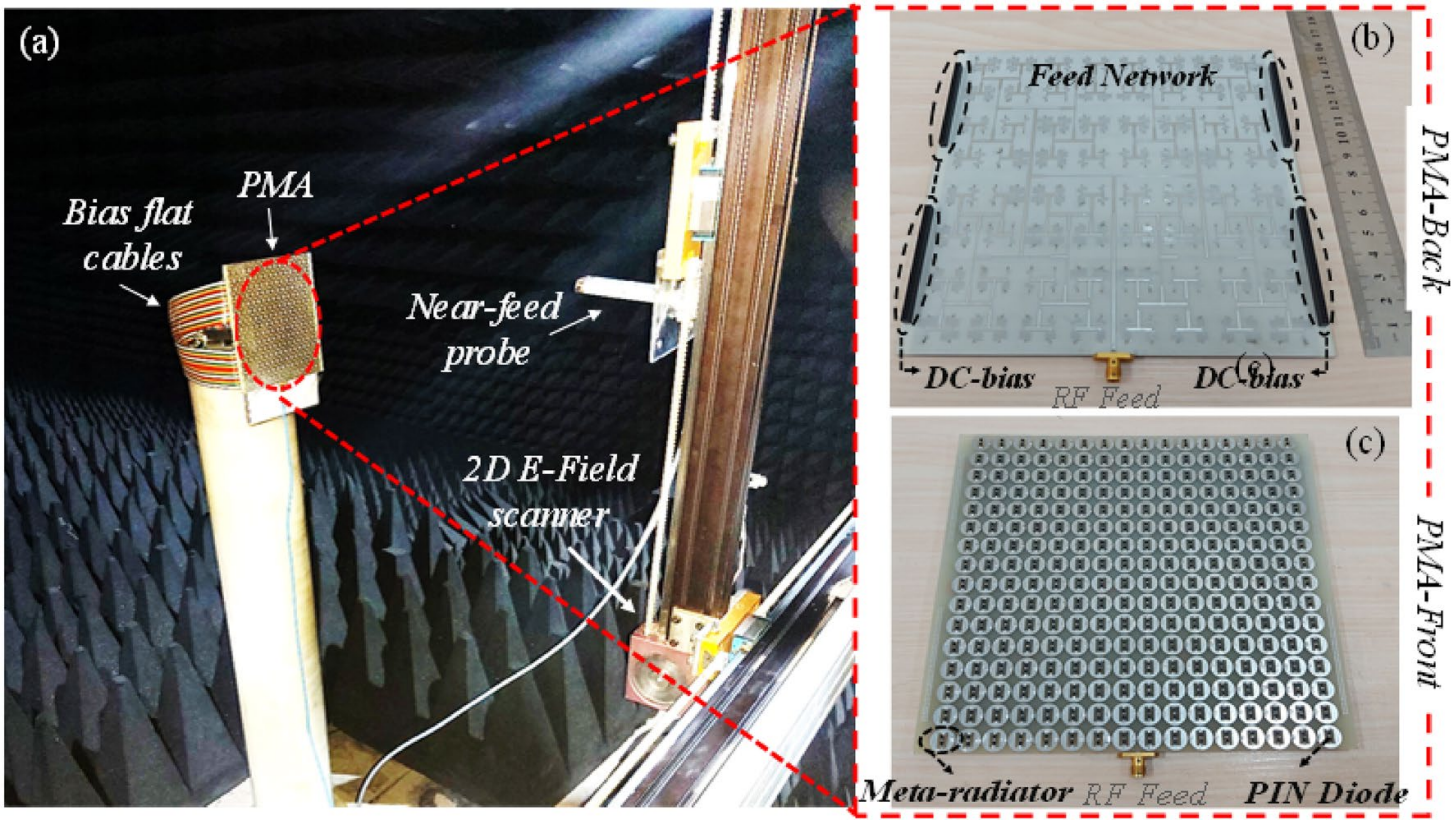
The prototype antenna for an OAM near-field measurement system: (**a**) anechoic chamber with a planar near-field scanning pattern. (**b**) Bottom view of the fabricated PMA. (**c**) Top view of the fabricated PMA.

**Figure 7:**
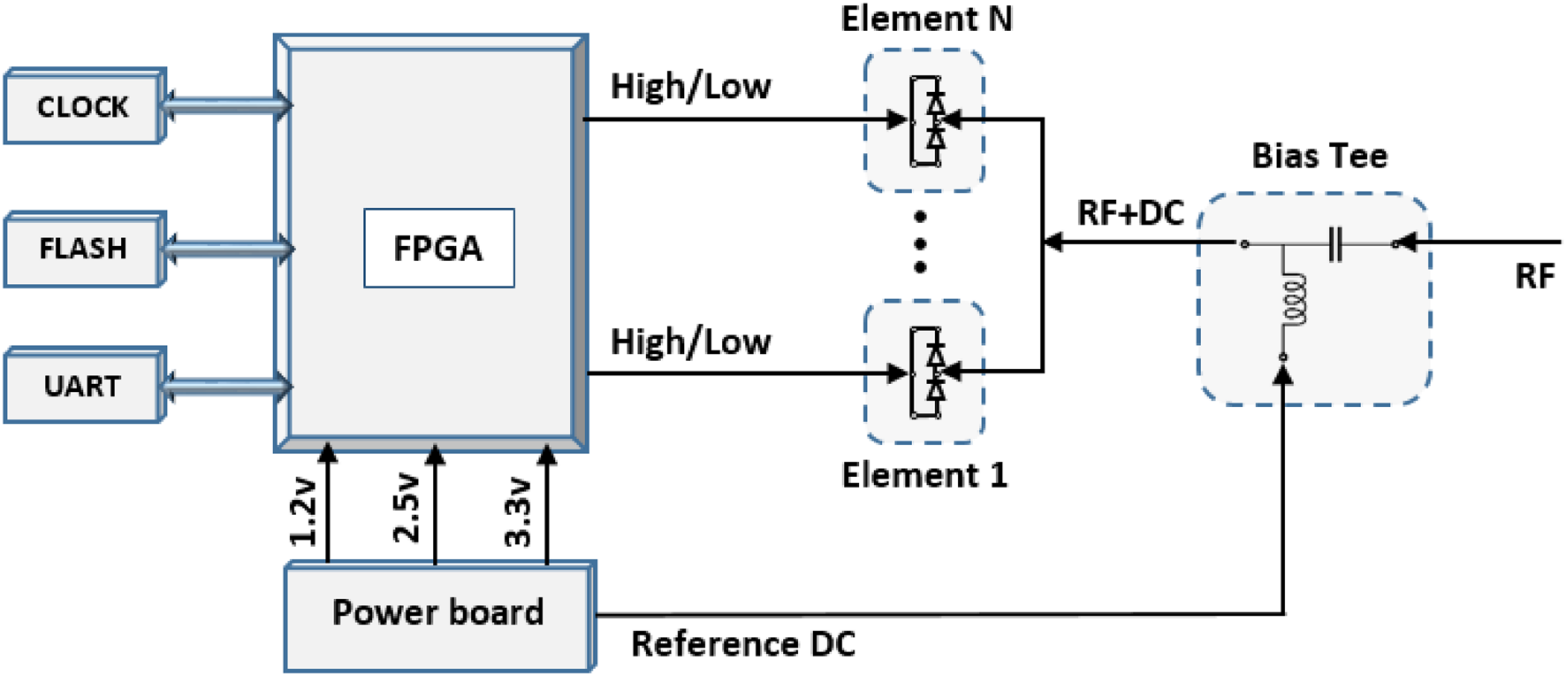
A block diagram of the steering-logic board circuit design.

As a means of further inspecting and validating the actual functionality of the proposed programmable metasurface antenna for generating dynamical multi-mode mm-Wave OAM beams, a prototype antenna consisting of a $16\times 16$ binary meta-radiators was fabricated based on multilayer PCB (printed circuit board) manufacture process. Figure 6b, c show the back and front views of the fabricated prototype for the programmable metasurface antenna with dimension of $160 \mathrm{mm}\times 160 \mathrm{mm}$. The array consists of 512 PIN diodes (SMP 1340), where the programmable-logic board controls each metal-radiator through 256 independent bias line. All bias lines are terminated to four 2 × 60 pins 1.27 mm through hole technology connectors, as shown in Fig. 6b. An external power supplies with 24V voltage inputs power the programmable logic board, which provides high and low bias voltages to the meta-radiators. The schematic diagram of the control board and array antenna are shown in Fig. 7, in which the main processing system uses a field programmable gate array (FPGA) to distribute the high and low biasing voltage, simultaneously across all of the units within the assigned clock signal based on the designed quantized phase distributions. Additionally, the control board provides an external reference voltage, which is mixed with an RF signal for comparison with the biasing voltages. The array is excited by corporate feed network, which terminates to 50 Ω Sub Miniature Version A (SMA) connector and was printed on a Rogers 4003 dielectric $\left({\varepsilon }_{r}=3.55, tan\delta =0.0027\right)$ with the thickness of 0.5 mm.

**Figure 8:**
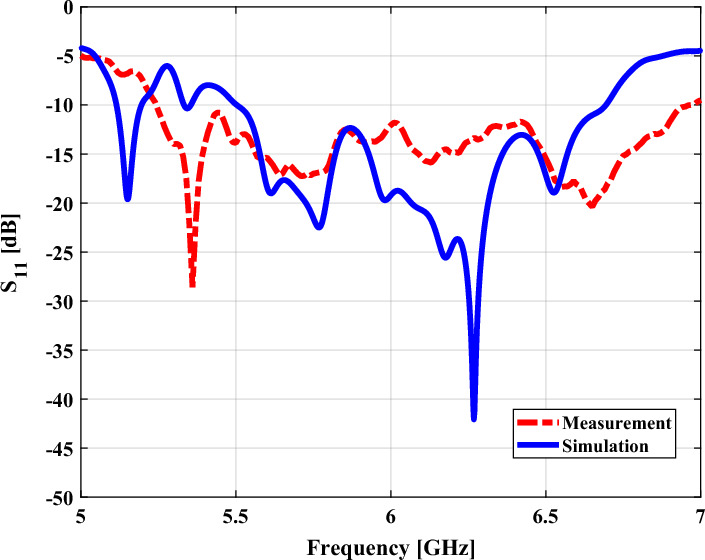
Simulated and measured reflection coefficients of the programable metasurface antenna.

The return loss (|S11|) of the fabricated programmable metasurface antenna was measured with the VNA, as shown in Fig. 8. According to the comparison of the experimental and simulation results, the measurement result achieves wider impedance bandwidth than the simulation one, which is resulted by the higher loss of the FR-4 substrate in fabrication. However, the measured and simulated results are in reasonable agreement.

A near-field scanning method is used to determine the amplitude and phase distributions of electric fields in the near region of antenna, as shown in Fig. 6a. In order to detect the wavefront of the OAM beam, a planar surface 18 wavelength from the metasurface antenna is discretized and scanned over a range of 250 mm × 250 mm. Near-field domain criteria are ensured by selecting such a distance between the programmable metasurface antenna and probe. In the experiments, the vertical polarization component of the radiated E-field is measured by using an open-ended waveguide antenna.

**Figure 9:**
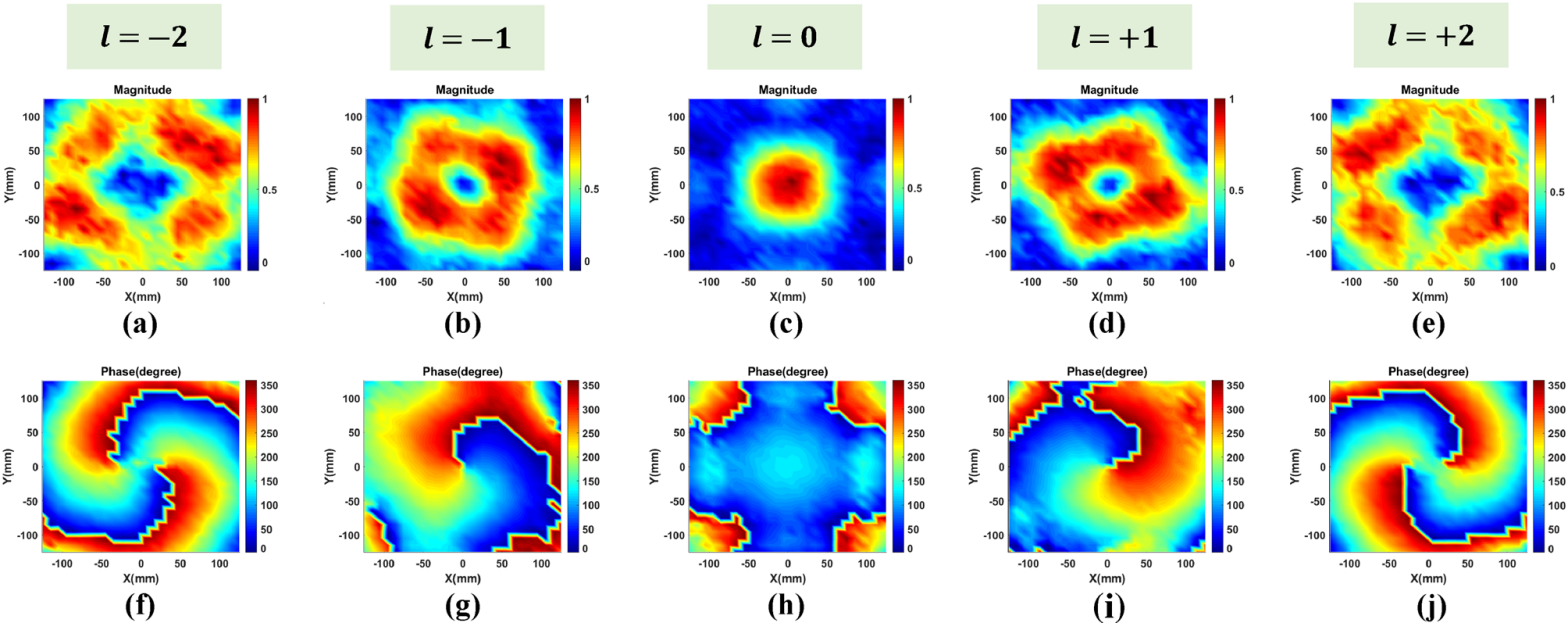
Measured magnitude and phase distribution of the electrical near-field on a planar surface which is positioned 18 wavelengths away from the metasurface antenna for different modes. Amplitude: (**a**) $l=-2$, (**b**) $l=-1$, (**c**) $l=0$, (**d**) $l=+1$ and (**e**) $l=+2$. Phase: (**f**) $l=-2$, (**g**) $l=-1$, (**h**) $l=0$, (**i**) $l=+1$ and (**j**) $l=+2$.

Figure 9 shows the normalized amplitude and phase distributions of the vertical component electric field measurements for the four modes $l=-2$, $l=-1$, $l=0$, $l=+1$, and $l=+2$. In line with expectations, all magnitude distributions for the nonzero OAM mode show a circular-ring shape with a null in the center, which represents one of the distinctive characteristics of beams that carry OAM. In addition, 360° helical phase distribution can already describe the OAM beam generation by using a 1-bit phase element. It is also possible to realize OAM modes with different helical directions in both negative and positive direction. It should be noted that experimental results vary slightly compared with the simulation ones, because of measurement units coupling, manufacturing tolerances, and the testing environment.

In most cases, OAM beams are typically generated by continuous phase variations, but the 1-bit programmable metasurface antenna presented in this paper only has binary phases, which may have a negative impact on its efficiency and mode purity when used in OAM generation. In spite of this, we are able to demonstrate that even a 1-bit programmable metasurface antenna can generate multimode OAM beams with a high degree of effectiveness and efficiency.

The mode purity of an OAM beam generator is one of the most important factors when evaluating its performance. According to Fourier transforms, the Fourier relationship between ${A}_{l}$ and $\psi \left(\varphi \right)$ can be describe as:

4 $${A}_{l}=\frac{1}{\sqrt{2\pi }}{\int }_{-\pi }^{\pi }\psi \left(\varphi \right)exp\left(-jl\varphi \right)d\varphi$$

in which ${A}_{l}$ is the amplitude of the OAM states while φ is the azimuthal angle.

The $\psi \left(\varphi \right)=Aexp\left(j\varphi \right)$ represents the sampling value along the ring with maximum amplitude in the nearfield sampling plane, A is the amplitude value and φ is the phase value. Then, a mode purity for mode L can be calculated as follows

5 $${Purity}_{l}=\frac{{\left|{A}_{l}\right|}^{2}}{\sum_{l=-\infty }^{l=\infty }{\left|{A}_{l}\right|}^{2}}$$

**Figure 10:**
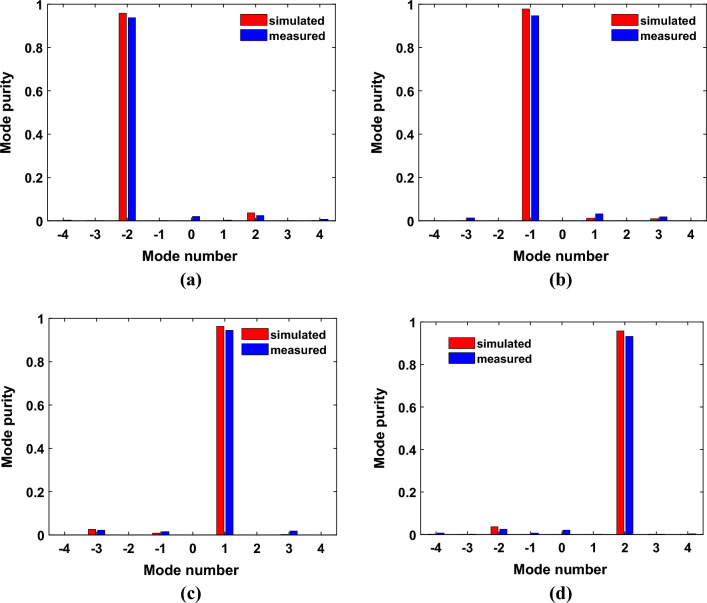
Simulated and measured OAM purity at frequency of 6GHz for different mode: (**a**) $l=-2$, (**b**) $l=-1$, (**c**) $l=+1$, (**d**) $l=+2$.

According to Eqs. (4) and (5), a comparison of the mode purity between simulations and measurements can be seen in Fig. 10 for the proposed PMA. In comparison with parasitic OAM modes, desired OAM modes have a relatively dominant position.

**Figure 11:**
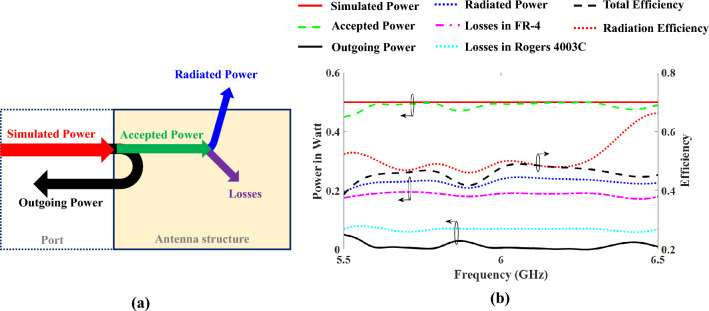
(**a**) Power balance of the antenna. (**b**) Power and efficiency at operating frequency at proposed PMA.

Some parameters impact on antenna efficiency such as including power outgoing from antenna ports, mismatches between antenna and RF front end circuits, and losses in substrate materials used, including FR-4, antenna layer, and Rogers 4003C, and RF feeding layer. The Fig. 11a illustrates the overall power balance of the proposed antenna to help understand its behavior. A power of 0.5 W was applied to the antenna in stimulation, as shown in Fig. 11b. When this power injects into the antenna, depending on its match, some of the power is reflected and returned to the source called "Outgoing Power". In this structure, the outgoing power is negligible due to the good input impedance matching at operating frequency. Remaining power enters into the antenna structure called "Accepted Power", which dives into parts known as "Radiated Power" and "Losses". At operating frequency, the accepted power is near with the stimulated one. The FR-4 substrate results the most loss, approximately 0.2 W, due to its larger loss tangent than the Rogers 4003C substrate. The antenna total efficiency is defined as the ratio between the radiated power and the stimulated one which is 50% at its operating frequency band. The antenna radiation efficiency is defined as the ratio between the radiated power and the accepted power. Notice that the power and efficiency curves are presented in one figure (Fig. 11b) which are meaningful based on the left and right axes of the figure, respectively.

Additionally, a comparison of the overall performance of the proposed metasurface antenna with the state of the art references is shown in Table 1. Aside from the high aperture efficiency relative to the occupied space, the proposed OAM radiation is produced by using a planar structure. Clearly, this structure represents a viable, capable and valuable alternative for future hybrid communications combining mm-wave with multi-mode OAM that are space-constrained.

**Table 1:** Comparison of the proposed PMA with state-of-the-art references.

Mode of operation/Ref	Phase resolution (bit)	f_0_ (GHz)	Number of layers	Dimension (λ_0_)	Realized gain @ $\left(l=0\right)$ (dB)	Aperture efficiency (%)
Reflection^17^	1	7.5	4	10 × 10 × 6	24.2	20.9
Transmission^35^	1	29	4	10 × 10 × 5	25.68	29.4
Radiation/this paper	1	6	4	3.2 × 3.2 × 0.05	15.9	30.2

## Conclusion

This paper established a new paradigm for the generation of multi-mode OAM-beams by using programmable metasurface antennas that extends beyond the scope of fixed-mode metasurface antennas. By using the anti-symmetry configuration between the two PIN diodes embedded with the unit, nearly the same electric field magnitudes but inverse phase states were obtained at the operating frequency, 6 GHz. The multimode OAM beams were achieved by using real-time programmable multimode metasurface antennas and dynamical modulation of quantized phase distributions. The proposed structure demonstrated the capability to increase the channel capacity and improve spectrum efficiency by independently multi-beaming across different channels, simultaneously. As a proof-of-concept, a digital structure was manufactured to demonstrate the remarkable capabilities of the programmable metasurface antenna. The simulation and measurement results are in good agreement with theories where demonstrate the effectiveness of the proposed PMA. This paper presents new methods and results for time-division multiple access in cellular communications, based on the generation of structured beams.

## Data Availability

The data are available from the authors upon reasonable request to S.H. Sedighy via email sedighy@iust.ac.ir.
